# Effects of ultraviolet treatment and alendronate immersion on osteoblast-like cells and human gingival fibroblasts cultured on titanium surfaces

**DOI:** 10.1038/s41598-019-39355-3

**Published:** 2019-02-22

**Authors:** Changjoo Jeon, Kyung Chul Oh, Kyu-Hyung Park, Hong Seok Moon

**Affiliations:** 1 Department of Prosthodontics, College of Dentistry, Yonsei University, Seoul, 03722 Korea; 2 Department of Prosthodontics, Oral Science Research Center, BK21 Plus Project, College of Dentistry, Yonsei University, Seoul, 03722 Korea

## Abstract

In this study, we evaluated the effects of ultraviolet (UV) treatment and alendronate (ALN) immersion on the proliferation and differentiation of MG-63 osteoblast-like cells and human gingival fibroblasts (HGFs) cultured on titanium surfaces. MG-63 cells were used for sandblasted, large grit, and acid-etched (SLA) titanium surfaces, and HGFs were used for machined (MA) titanium surfaces. SLA and MA specimens were subdivided into four groups (*n* = 12) according to the combination of surface treatments (UV treatment and/or ALN immersion) applied. After culturing MG-63 cells and HGFs on titanium discs, cellular morphology, proliferation, and differentiation were evaluated. The results revealed that UV treatment of titanium surfaces did not alter the proliferation of MG-63 cells; however, HGF differentiation and adhesion were increased in response to UV treatment. In contrast, ALN immersion of titanium discs reduced MG-63 cell proliferation and changed HGFs into a more atrophic form. Simultaneous application of UV treatment and ALN immersion induced greater differentiation of MG-63 cells. Within the limitations of this cellular level study, simultaneous application of UV treatment and ALN immersion of titanium surfaces was shown to improve the osseointegration of titanium implants; in addition, UV treatment may be used to enhance mucosal sealing of titanium abutments.

## Introduction

The placement of dental implants has become an essential method for the treatment of edentulous or partially edentulous patients. Titanium remains the material of choice for dental implants because of its superior biocompatibility and mechanical strength^1^. It spontaneously forms a dense titanium dioxide (TiO_2_) layer at its surface when exposed to air or aqueous electrolytes, which acts as a strong barrier against corrosion and ion release from the metal surface, contributing to high biocompatibility^2,3^. Moreover, this oxide layer enhances protein adsorption after implantation and consequently affects osseointegration, which is defined as “the direct structural and functional connection between ordered, living bone, and the surface of a load-carrying implant”^4–6^.

Dental implants consist of an implant fixture, an implant abutment, and an upper prosthesis. In general, implant fixtures have rough surfaces for enhanced osseointegration, whereas implant abutments are composed of smooth or machined surfaces for prevention of biofilm formation^7^. However, for tissue-level implants, the implant fixtures partly consist of machined surfaces on the coronal portion, while the other portion consists of rough surfaces; hence, two types of surface characteristics co-exist in these implants.

Numerous reports have shown that modifications to the microgeometry and roughness of implant surfaces contribute to the achievement of better osseointegration^8^. Examples of these modifications include subtractive methods, such as blasting, etching, and oxidation, and additive methods such as titanium plasma spraying^9^. However, a drawback resulting from the inherent properties of the titanium material itself still remains: the bioactivity of titanium substantially decreases over time when exposed to air due to the appearance of hydrocarbons, resulting in loss of hydrophilicity^10^. This contamination of titanium surfaces has been shown to be related to the initial affinity for human osteoblasts, causing reduced migration and attachment^11^. A common strategy used to convert the hydrophobic titanium surface to the hydrophilic state was the use of ultraviolet (UV) irradiation, whose effects in this context were reported in the late 1990s^12^. UV-treated titanium surfaces not only exhibited alteration of physiochemical properties, but also showed improvement of biologic capabilities, such as increased cell proliferation and enhanced osteoblast differentiation; this phenomenon was termed as photofunctionalisation^13^.

Bisphosphate application is another method used to increase osteoblastic activity. In contrast to UV treatment which indirectly affects osteoblast functions, bisphosphates affect osteoblastic activity in a more direct manner. Although bisphosphonates have been generally used as antiresorptive agents for bone-related disorders by inhibiting osteoclastic activity^14^, several recent studies have reported that these compounds may increase osteoblastic activity^15–18^. Alendronate (ALN), a type of nitrogen-containing bisphosphonates that is currently one of the most effective agents for treating various bone diseases, increases alkaline phosphatase (ALP) activity and promotes the expression of genes encoding bone morphogenic protein-2, type I collagen, and osteocalcin, indicating its ability to induce proliferation and maturation of osteoblasts^19–23^. However, its application to titanium implants has shown varying results, as is also the case with the use of bisphosphonates in such implants^24,25^. In order to avoid potential complications from systemic application, several attempts have been made to administer alendronates locally and therefore to restrict ALN-induced osteoblastic activity to peri-implant bone regions^26,27^. A recent study suggesting the concurrent application of UV irradiation and ALN soaking to the titanium surfaces represents an example of such attempts^28^.

The long-term success of implant treatments requires not only the osseointegration of implant fixtures but also a rigid and tight seal around the implant abutments. Peri-implant soft tissues prevent subgingival biofilm formation through proper sealing and exhibit the potential to defend against bacterial penetration; these properties are important for implant maintenance and oral hygiene control^7,29–31^. Several studies have been carried out to improve the adhesion of soft tissue on implant abutment surfaces^32–34^. However, limited number of studies have examined the effects of UV treatment on the relationship between the machined titanium surface and soft tissues; one study showed that the proliferation of human gingival fibroblasts (HGFs) was significantly increased depending on the thickness of TiO_2_ in TiO_2_-coated titanium surfaces when UV irradiation was performed^35^.

Considering that implant abutments are in contact with soft tissues and are composed of machined surfaces, it is necessary to investigate the effects of surface treatment methods on machined surfaces and demonstrate the responses of HGFs. Moreover, when aiming to apply either or both surface treatment methods to tissue-level implants that have both machined and rough surfaces, it is crucial to determine the effects of surface treatment methods on the fibroblast response. However, to our knowledge, there are no reports describing the effects of ALN immersion on HGFs cultured on machined titanium surfaces. Furthermore, as simultaneous application of UV irradiation and ALN immersion had been conducted in osteoblast-like cells in a previous study, it is reasonable to apply similar approaches to the HGFs.

Hence, we aimed to evaluate the effects of UV treatment and ALN immersion on the responses of osteoblast-like cells cultured on titanium discs with rough surface, and to assess the effects of these treatments on the responses of HGFs cultured on titanium discs with machined surface. The experiments were divided into the investigation of two types of surfaces to resemble clinical situations: titanium discs with rough surfaces were used to represent implant fixtures and those with machined surfaces were used to represent implant abutments. Osteoblast-like cells were used to evaluate hard tissue reactions on titanium surfaces at the implant fixture level, while HGFs were used to assess soft tissue reactions on titanium surfaces at the implant abutment level. The effects of simultaneous application of UV irradiation and ALN immersion on the responses of osteoblast-like cells and HGFs were also investigated.

## Materials and Methods

### Preparation of titanium specimens

Titanium specimens were prepared in the shape of discs (10-mm diameter and 2-mm thickness) from commercially pure grade IV titanium (Dentium Co., Suwon, Korea). One half of the specimens were in the sandblasted, large grit, and acid-etched (SLA) state, and the other half was left untreated, i.e., in the machined (MA) state. The SLA specimens were sandblasted with aluminium oxide (Al_2_O_3_) and acid-etched with hydrochloric acid (HCl), and were then used to evaluate the responses of osteoblast-like cells. The MA specimens were ultrasonically cleaned in soap solution for 4 h, rinsed in distilled water, dried with oil-free air, and used to evaluate the reaction of HGFs. The SLA specimens were subdivided into four groups (*n* = 12 for each group); the discs in the S group received no further surface treatment, those in the SUV group were treated with UV irradiation, those in the SAN group were immersed in ALN, and those in the SUVAN group received both surface treatments. The MA specimens were also subdivided into four groups (*n* = 12 for each group) in a similar manner as the SLA specimens, replacing S with M in the group names. In total, 96 titanium discs were used in this study (Table 1). All specimens were sealed individually and sterilised by gamma irradiation.

**Table 1:** Classification of the experimental design. S and M groups served as control groups for each cell line.

Group	S	SUV	SAN	SUVAN	M	MUV	MAN	MUVAN
Basic surface	SLA surface				Machined surface			
UV	X	O	X	O	X	O	X	O
ALN	X	X	O	O	X	X	O	O
Cell line	MG-63 human osteoblast-like cells				Primary gingival fibroblasts (HGF)			

### UV treatment of titanium surfaces

For UV-treated groups (SUV, SUVAN, MUV, and MUVAN), the specimens were exposed to UV radiation for 15 min under ambient conditions using a UV-light-emitting device (TheraBeam SuperOsseo; Ushio Inc., Tokyo, Japan) 24 h before cell culture. The UV light was delivered as mixed spectrum through single UV lamp at wavelengths of 360 and 250 nm^36^.

### ALN immersion of titanium surfaces

Specimens from the SAN, SUVAN, MAN, and MUVAN groups were immersed in a solution of 10^−3^ M ALN (Cayman Chemical, Ann Arbor, MI, USA) for 24 h. For the SUVAN and MUVAN groups, in which both surface treatment methods were applied, UV photofunctionalisation procedures preceded ALN immersion.

### Cell culture

MG-63 human osteoblast-like cells (KCLB No. 21427) were purchased from the Korean Cell Line Bank (KCLB, Seoul, Korea), and primary gingival fibroblasts (PCS-201-018) were purchased from the American Type Culture Collection (ATCC, Manassas, VA, USA). The cells were seeded on 10-cm tissue culture dishes in standard cell culture medium consisting of Dulbecco’s modified Eagle’s medium (Invitrogen, Waltham, MA, USA) containing 10% foetal bovine serum (Invitrogen) and antibiotics (50 U/mL penicillin G and 50 μg/mL streptomycin; Invitrogen). Samples were incubated at 37 °C in a 5% CO_2_/95% air atmosphere at 100% relative humidity. The cell culture medium was changed every 2–3 days. When the cells reached 85–90% confluence, they were treated with a trypsin-ethylenediaminetetraacetic acid solution (Invitrogen), resuspended in culture medium, and seeded in new dishes at a 1/5 dilution.

### Scanning electron microscopy (SEM)

MG-63 cells and HGFs were used for SLA and MA discs, respectively and seeded at 1 × 10^4^ cells/disc on the surface of each specimen in a 24-well plate. After incubation for 24 h, a specimen from each of the eight groups was prefixed with Karnovsky’s fixative (2% glutaraldehyde-paraformaldehyde; Invitrogen) in 0.1 M phosphate buffer (PB, pH 7.4) for 6 h and washed twice for 30 min in 0.1 M PB. The specimens were postfixed with 1% osmium tetraoxide dissolved in 0.1 M PB for 1.5 h, and washed with 0.1 M PB for 10 min. Specimens were then dehydrated in increasing concentrations of ethanol (50%, 60%, 70%, 80%, 90%, 95%, and 100%), infiltrated with isoamyl acetate, and subjected to critical point drying (Leica EM CPD300; Leica Mikrosysteme GmbH, Vienna, Austria). The specimens were coated with platinum (5 nm thickness) using an ion coater (Leica EM ACE600; Leica Mikrosysteme GmbH) and evaluated by field-emission SEM (Merin; Carl Zeiss, Jena, Germany). Representative images were captured at specific magnifications (500x, 2000x).

### Proliferation assay

2-(2-Methoxy-4-nitrophenyl)-3-(4-nitrophenyl)-5-(2,4-disulfophenyl)-2H-tetrazolium, monosodium salt (WST-8) assays were used to assess cell proliferation. First, six SLA or MA discs from each group were distributed in 24-well plates. The cells were trypsinised, resuspended in culture medium, and seeded in 24-well plates on SLA or MA discs at 1 × 10^4^ cells/disc. Cells were then cultured for 4 h at 37 °C in an atmosphere containing 5% CO_2_. The proliferation of MG-63 cells and HGFs was measured using Cell Counting Kit-8 (CCK-8; Dojindo, Kumamoto, Japan) according to the manufacturer’s instructions. The cell suspensions in each group were inoculated into 96-well plates, and 10 μL of the CCK-8 solution was added to each well of the plate, being careful to avoid the formation of bubbles. After incubating the plate for 1 h, the optical density (OD) of the water-soluble, yellow-coloured formazan, WST-8, formed by vital cells in each well was measured at 450 nm using an automated microplate reader (VersaMax Tunable microplate reader; Molecular Devices Co., Sunnyvale, CA, USA).

### ALP activity assay

Differentiation of MG-63 cells was assessed by evaluating ALP expression using a QuantiChrom ALP Assay Kit (BioAssay Systems, Hayward, CA, USA). Five specimens from each of the four SLA groups (S, SUV, SAN, and SUVAN) were placed in 24-well plates; 1 × 10^4^ cells were seeded on each specimen and cultured for 3 days at 37 °C in an atmosphere containing 5% CO_2_. Working solutions/reagents were prepared for each 96-well assay with 200 μL assay buffer, 5 μL magnesium acetate, and 2 μL *p*-nitrophenyl phosphate. Cells seeded on the discs were washed with phosphate-buffered saline (Invitrogen) and then lysed in 0.5 mL of 0.5% Triton X-100 for 20 min. Next, 5 μL of cell lysate mixed with 195 μL working solution was transferred to each well of the 96-well plate, and the ODs of each well were read at 405 nm at 0 and 4 min on a plate reader. ALP activities of the sample (IU/L = μmol/[L × min]) were calculated using the formula below:

$$=\,\frac{(O{D}_{Samplet}-O{D}_{Sample0})\times 1000\times Reaction\,Vol}{t\times \varepsilon \times l\times Sample\,Vol}=\frac{(O{D}_{Samplet}-O{D}_{Sample0})\times 1000\times Reaction\,Vol}{(O{D}_{Calibrator}-O{D}_{{H}_{2}O})\times Sample\,Vol\times t}\times 35.3$$

### Real-time reverse transcription polymerase chain reaction (RT-PCR) analysis

To evaluate the differentiation of HGFs, mRNA levels of integrin-β1, type I and III collagens, fibronectin, and laminin5 were analysed by RT-PCR. HGFs were cultured at 1 × 10^4^ cells/disc for 24 h on five discs from each of M, MUV, MAN, and MUVAN groups, and total RNA was isolated with a Hybrid-R kit (GeneAll Biotechnology Co., Ltd., Seoul, Korea). cDNA was synthesised using a Maxime RT Premix kit (iNtRON Biotechnology, Sungnam, Korea) according to the manufacturer’s recommendations. Gene expression analysis was carried out using a SensiFAST SYBR Hi-ROX Kit (Bioline USA Inc., Taunton, MA, USA) on an ABI StepOnePlus Real-Time PCR machine (Applied Biosystems, Foster City, CA, USA). The specific amplification primers were from Integrated DNA Technologies (Coralville, IA, USA; Table 2).

**Table 2:** Lists of primer sequences used for RT-PCR analysis in this study.

	Forward primers (5′ → 3′)	Reverse primers (5′ → 3′)	Product length (bp)
Integrin-β1	TGTAAGGAGAAGGATGTTGACG	CAACCACACCAGCTACAATTG	142
Type I collagen	CCCCTGGAAAGAATGGAGATG	TCCAAACCACTGAAACCTCTG	148
Type III collagen	AAGTCAAGGAGAAAGTGGTCG	CTCGTTCTCCATTCTTACCAGG	125
Fibronectin	ACTGTACATGCTTCGGTCAG	AGTCTCTGAATCCTGGCATTG	74
Laminin5	CAAATGTGACCAGTGCAGC	CATCCCTCCATATCCACGAAC	144

### Statistical analysis

The OD values measured by WST-8 assays and the results of ALP activity assays and RT-PCR were analysed by one-way analysis of variance. Bonferroni post-hoc tests were performed for pairwise comparisons of OD, ALP activity, and RT-PCR data. All statistical analyses were performed using IBM SPSS statistics software (version 23; SPSS Inc., Chicago, IL, USA) and the level of significance was set as α = 0.05.

## Results

### Cellular attachment and morphology of MG-63 cells and HGFs on titanium surfaces

**Figure 1:**
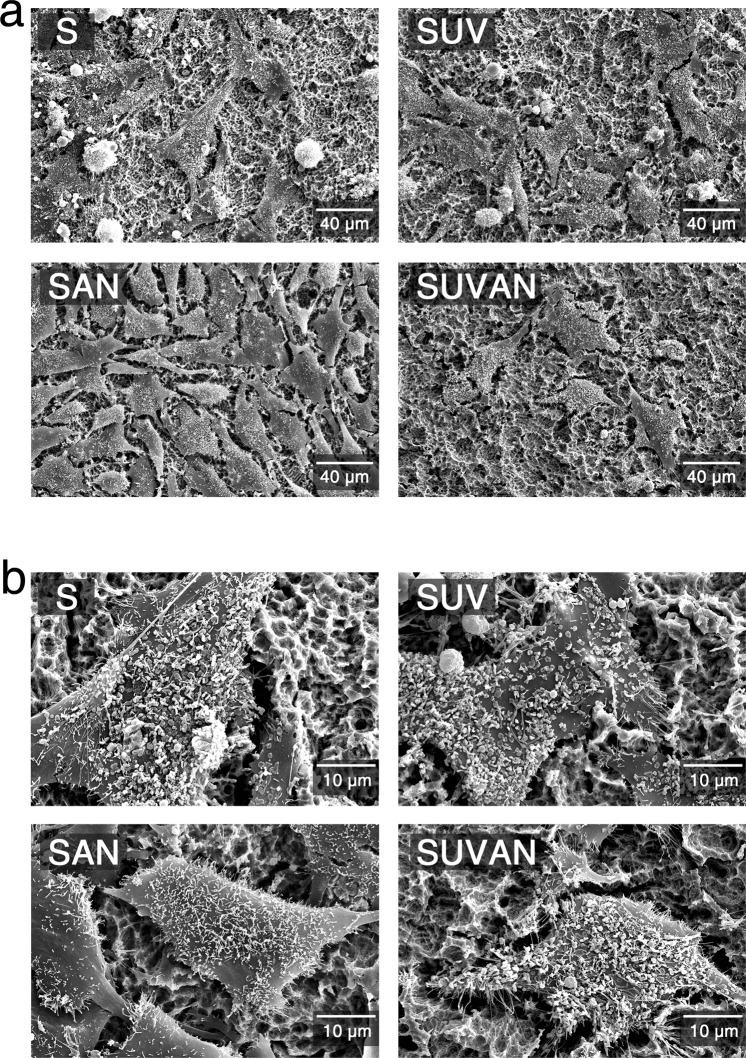
Representative SEM images of MG-63 cells cultured on titanium surfaces in the S, SUV, SAN, and SUVAN groups. (**a**) SEM images at 500x magnification, (**b**) SEM images at 2000x magnification.

**Figure 2:**
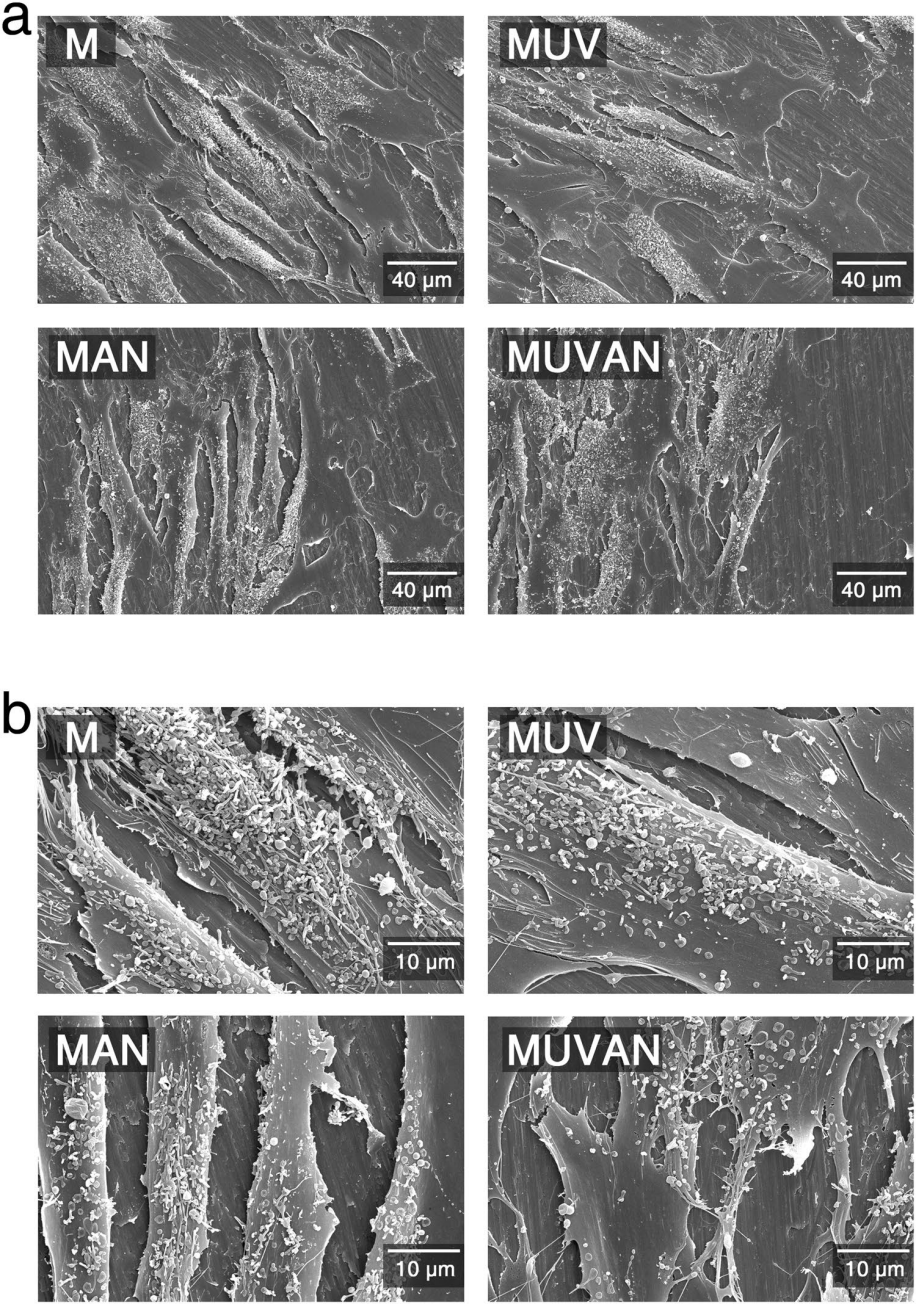
Representative SEM images of HGFs cultured on titanium surfaces in the M, MUV, MAN, and MUVAN groups. (**a**) SEM images at 500x magnification, (**b**) SEM images at 2000x magnification.

SEM was used to qualitatively assess cellular attachment and cell morphology on titanium surfaces. MG-63 cells on SLA discs (groups S, SUV, SAN, and SUVAN) grew normally, regardless of surface treatment methods applied, and actively adhered to the titanium surfaces (Fig. 1a). At a higher magnification (2000x; Fig. 1b), extracellular vesicles were observed. For HGFs grown on MA discs (groups M, MUV, MAN, and MUVAN), the cells grew in a concentric dispersion, following the lines on the titanium surfaces (Fig. 2a). When observed at a higher magnification (2000x; Fig. 2b), typical features of cell adhesion, such as cytoplasmic prolongation and filopodia, were observed. In the middle portion of the discs, the cellular attachment pattern appeared similar among groups, whereas around the border of the discs, MAN and MUVAN groups showed fewer exosomes and microfilaments, and the cells were thinner.

### Proliferation of MG-63 cells and HGFs

**Figure 3:**
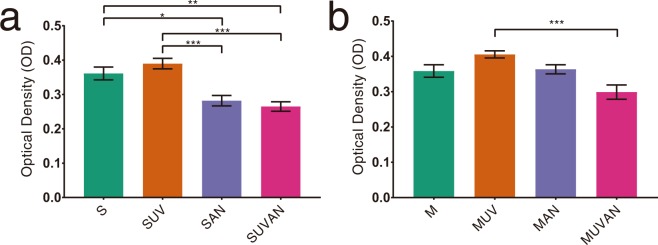
Effects of UV treatment and ALN immersion on cellular proliferation, as determined using WST-8 assays. Cellular proliferation was expressed as optical density (OD). (**a**) OD of MG-63 cells in the S, SUV, SAN, and SUVAN groups; (**b**) OD of HGFs in the M, MUV, MAN, and MUVAN groups. Data are expressed as means ± S.E.M. (*n* = 6). *P < 0.05, **P < 0.01, ***P < 0.001 for comparisons among groups. The asterisks indicate statistically significant differences between the groups.

After 4 h of cell culture, the OD values of the SAN and SUVAN groups were significantly lower than those of the S and SUV groups (*P* < 0.05; Fig. 3a). For HGFs, the MUV group showed a higher OD value than the M group, although the results were not statistically significant. Additionally, the MUVAN group showed the lowest value, which was significantly lower than that in the MUV group (*P* < 0.05; Fig. 3b).

### Differentiation of MG-63 cells

**Figure 4:**
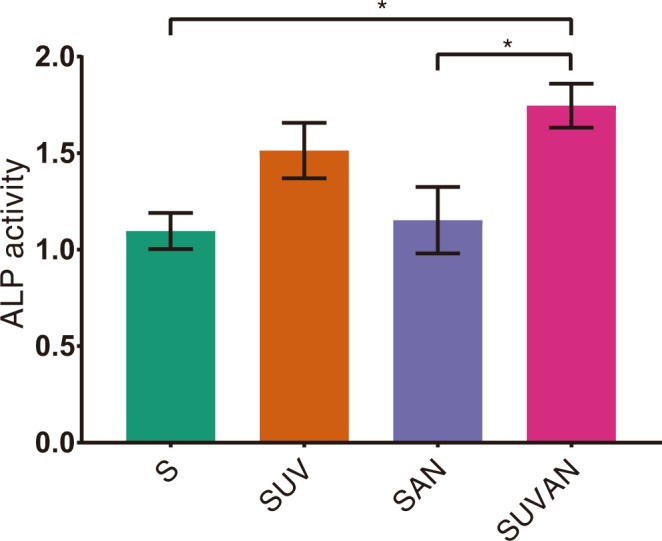
Effects of UV treatment and ALN immersion on ALP activity in MG-63 cells, as assessed by spectrophotometry after 3 days of culture, in the S, SUV, SAN, and SUVAN groups. Data are expressed as means ± S.E.M. (*n* = 5). *P < 0.05 for comparisons among the groups. The asterisks indicate statistically significant differences between the groups.

ALP activity in the S group did not differ from that in the SUV group. However, the SUVAN group showed significantly higher ALP activity than the S group (*P* < 0.05). The ALP activity of the SUVAN group was significantly higher than that of the SAN group (*P* < 0.05), but did not differ from that of the SUV group (*P* > 0.05; Fig. 4).

### Differentiation of HGFs

**Figure 5:**
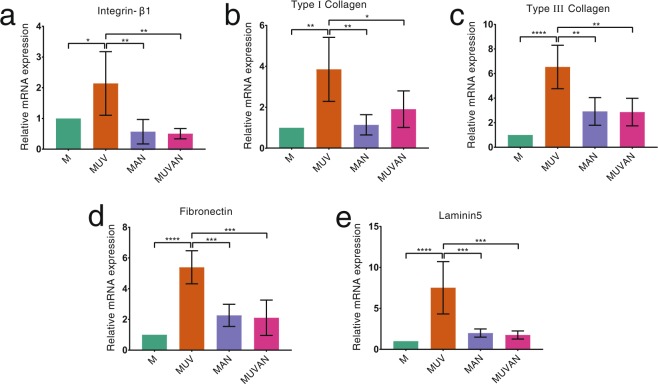
Effects of UV treatment and ALN immersion on cellular differentiation in HGFs in the M, MUV, MAN, and MUVAN groups. RT-PCR data indicate the relative mRNA expression of (**a**) integrin-β1, (**b**) type I collagen, (**c**) type III collagen, (**d**) fibronectin, and (**e**) laminin5 after 24 h of culture. Data are expressed as means ± S.E.M. (*n* = 5). *P < 0.05 for comparisons among the groups. The asterisks indicate statistically significant differences between the groups.

The mRNA levels of integrin-β1, type I and III collagen, fibronectin, and laminin5 in the MUV group were significantly higher than those in the M, MAN, and MUVAN groups (*P* < 0.05; Fig. 5). However, there were no statistically significant differences between the mRNA levels of all the target genes in the M, MAN, and MUVAN groups (*P* > 0.05; Fig. 5).

## Discussion

In order to improve the initial stability and achieve long-term success of implant treatments, numerous studies have been carried out to evaluate the biological responses on various kinds of implant surfaces at cellular levels^37,38^. As a result, it is generally accepted that rough surfaces are suitable for implant fixtures, whereas machined or turned surfaces are recommended for implant abutments^39^. However, various approaches to modify surfaces of the implants are still being developed to overcome a drawback resulting from the inherent properties of the titanium material itself^40,41^. The present study aimed to assess the potential utility of applying UV and/or ALN concurrently on both surfaces.

The SLA surface is manufactured by sandblasting with large grit particles, followed by acid-etching procedures with HCl/H_2_SO_4_, resulting in a rough surface^42^. All of the surface treatments applied to SLA surfaces showed favourable cell adhesion and maturation of extracellular matrix (ECM) in the present study, as shown by the SEM images, indicating that the surface treatments did not inhibit the growth and differentiation of MG-63 cells. The MG-63 cells in the SUVAN group exhibited significantly lower mitochondrial activity than those in the S group, as indicated by the cell proliferation assay^43^; however, they showed increased ALP activity relative to those in the S group. This can be explained by the proliferation/differentiation interrelationships during the progressive development of the osteoblast phenotype^44,45^. Osteoblasts undergo a sequential pattern of gene expression as differentiation progresses: (1) proliferation and ECM biosynthesis, (2) ECM development, maturation, and organisation, and 3) ECM mineralisation^44^. The ECM undergoes a series of changes in its configuration and structure during the immediate post-proliferative periods^44^. Further, as the mineralisation phase progresses, ALP activity shows early and progressively enhanced expression^44,46^. In the SAN group, the effects of ALN application alone on the response of MG-63 were unclear; in the SUV group, UV application alone did not elicit remarkable differences when compared with that in the S group. On the other hand, the concurrent application of UV treatment and ALN immersion facilitated the differentiation of MG-63 cells in the SUVAN group.

The synergistic effects described above can be explained from a methodological standpoint related to the loading protocol of ALNs on titanium surfaces. When delivering biomolecules onto the implant surfaces, several methods, such as adsorption, covalent immobilisation, or release from coatings, are available^47^. As methods to facilitate the loading of ALNs, the use of precoated hydroxyapatite layers^26^ or plasma treatment have been sugggested^27^. Another strategy is to apply UV irradiation: it has been hypothesised that UV irradiation of titanium surface causes the removal of surface hydrocarbons owing to the photocatalytic activity of TiO_2_, and that the resulting exposed Ti^4+^ sites attract ALN molecules. This is because ALNs are negatively charged at physiological pH^48,49^. This difference in charge enables ALNs to bind to the titanium surface more easily, and subsequently, facilitates the local apposition of ALNs through the hybridised mechanism of adsorption and release from coatings. The present study also demonstrated that the concurrent application of ALNs and UV treatment of SLA titanium surfaces resulted in an enhanced impact on osteoblast differentiation.

UV treatment of TiO_2_ surface causes the excitement of an electron from the valence band to the conduction band and creates a positive hole on the superficial layer^50^. This is followed by the transition process of electrons, causing catalysing chemical reactions. As a result, reactive oxygen species are produced at the surface of TiO_2_, which enables removal of hydrocarbon through the reaction of such radicals^51,52^. In addition to this principle, direct UV irradiation itself also affects dissociation of hydrocarbons^53^. Through these processes, the titanium surface becomes more hydrophilic and acquires positive charge transforming from its original electrostatic state into a surface with higher bioactivity, and exhibits enhanced protein adsorption and cellular adhesion^48^. However, there were no remarkable differences in the response of MG-63 cells between the SUV and S groups in the present study. Further studies under different culture conditions are required for clarification.

The contamination of titanium surfaces is attributed to the accumulation of hydrocarbon on titanium surfaces over time^11,53^. From a physicochemical point of view, titanium surfaces are known to continuously absorb organic impurities, such as polycarbonate and hydrocarbons, through the atmosphere, water, and cleaning liquids^54–56^. More importantly, the rate and capacity to absorb proteins are critical factors in evaluating the biocompatibility of implant surfaces^57^, and are directly correlated with the amount of surface carbons^11^. This was demonstrated by a previous study in which aged titanium surfaces showed substantially reduced capacity to absorb proteins, and the early albumin adsorption rate was reduced after 4 weeks of storage compared with that of fresh surfaces^10^. Considerable reduction in fibronectin adsorption ability was also detected, although the degree of decomposition was lower than that of albumin^10^. Moreover, aged titanium surfaces were more hydrophobic than the fresh titanium surfaces^53,58^.

Fibroblasts use integrin receptors for their attachment; integrin-β1 is an essential binding unit that enables the binding of fibroblasts to titanium surfaces^59^. Type I and III collagens are important constituents of ECM from healthy human gingiva^60^. Fibronectin and laminin5 are internal components of basement membrane and are proteins that contribute to cellular attachment^61^. Hence, we aimed to measure relative mRNA expression of the genes that encode these proteins. For the MUV group, mRNA expression of all the target genes exhibited significantly higher values compared with those of the other three groups, indicating that the photocatalytic activity of TiO_2_ may also affect the attachment of fibroblasts on machined titanium surfaces^35^. Several attempts have been made to promote fibroblast attachment onto titanium surfaces^62–64^. However, to our knowledge, the present study is the first to use UV irradiation as a variable in evaluating its effect on fibroblasts cultured on machined titanium surfaces. Hence, as a technique to improve the long-term prognosis of implants, UV treatment of titanium abutment surfaces is worthy of further validation via *in vivo* studies.

In contrast, the role of ALN on fibroblasts requires further exploration; the SEM images exhibited atrophic and inactive morphology of HGFs in the ALN-treated groups (MAN and MUVAN). Previous studies also have shown that bisphosphonates had negative effects on oral mucosal cells^26,65–67^. However, the present study did not show significant differences in fibroblast attachment between the M and MUVAN groups. This result is in contrast with the results derived from SLA surfaces, in which concurrent application of UV treatment and ALN immersion showed synergetic effects. This may be attributed to the differences between machined and rough titanium surfaces; a previous study reported that the effects of soaking on machined titanium discs were less pronounced than those on rough titanium surfaces^68^. Hence, concurrent application of UV irradiation and ALN immersion on machined titanium surfaces appears to have little effect on fibroblasts.

There are several limitations to the present study. In the experiments using MG-63 cells, the discs of the SLA surface were used to represent implant fixtures. However, because the SLA surface itself has been shown to have a high success rate with regard to osseointegration of dental implants, differences from other surface treatment methods may not be conspicuous^8,69^. In addition, the disc-shaped material differed from the clinical environment. We used the commercially available MG-63 cell line in this study, which shows osteoblastic activities with high levels of ALP activity and osteocalcin production during differentiation^70,71^. Although MG-63 cells have characteristics comparable to human osteoblasts, further experiments using human osteoblasts are needed. Additionally, we applied ALN at a concentration of 10^−3^ M. When MG-63 cells were exposed to 10^−8^ to 10^−6^ M ALN, the cellular activity of MG-63 cells was not altered. In contrast, 10^−3^ to 10^−2^ M ALN inhibited matrix metalloproteinases-2 activity in MG-63 cells^72^, and 10^−4^ M ALN promoted the secretion of pro-inflammatory cytokines from human osteoblasts and reduced proliferation and differentiation^73^. These results were obtained by direct application of ALN solution. As we only immersed titanium discs in ALN, further studies are needed to identify the optimal ALN concentration for immersion.

It is necessary to distinguish between the effects of these surface treatments on the implant fixtures and implant abutments in order to apply these methods appropriately in clinical practice. For example, machined portion of the tissue-level implants, or implant abutments may not benefit from the concurrent application of UV irradiation and ALN immersion. Instead, it may be more reasonable to apply UV treatments on them. Moreover, the duration of UV treatment on the titanium surface may be an important issue with regard to implant abutments. In a recent study, the effects of UV treatment on the titanium surface were significantly reduced when the titanium was exposed to air for 28 days^74^. The procedures for dental prosthesis placement involve exposure of the abutments to air after they are fabricated; hence, it may be beneficial to apply UV treatment to abutments immediately before implant-abutment connection. Accordingly, a protocol to promote the early induction of mucosal sealing of the abutment by UV treatment may be designed. Further studies are needed to determine the duration for which these effects can be maintained and the effectiveness of this approach.

In summary, the present *in vitro* study highlights the feasibility of applying UV irradiation and ALN immersion as a strategy for the surface treatment of titanium discs. The results of this study extend our knowledge base to further develop better technologies for the surface treatment of titanium implant fixtures and abutments. The study showed a significant improvement in osteoblastic activity by simultaneous application of UV treatment and ALN immersion to titanium discs with rough surfaces; however, the individual application of each method did not yield significant enhancement. With regard to titanium discs with machined surfaces, concurrent application of UV irradiation and ALN immersion did not increase fibroblast attachment. However, UV treatment of machined titanium surfaces was found to be effective for enhancing fibroblast attachment. Further *in vivo* studies are necessary for detailed evaluation of the effects of these surface treatment methods for titanium implants and abutments in animal models.

## Data Availability

The authors declare that all data supporting the findings of this study are available within the article or from the corresponding author upon reasonable request.
